# Intravaginal immunization using a novel antigen delivery device elicits robust vaccine antigen-specific systemic and mucosal humoral immune responses

**DOI:** 10.1186/1742-4690-9-S2-P191

**Published:** 2012-09-13

**Authors:** PF McKay, JF Mann, A Pattani, VL Kett, K Malcolm, RJ Shattock

**Affiliations:** 1 Imperial College, London, UK; 2 School of Pharmacy, Queen's University, Belfast, UK

## Background

While it is relatively easy to elicit antigen-specific serum antibody it is much more difficult to establish meaningful levels of specific antibody at mucosal surfaces, the major route of viral invasion. We sought to determine if mucosal vaccination using topical vaginal application could initiate local antigen-specific immunity, enhance previously existing systemic immunity or re-target responses to the mucosae.

## Methods

We used a silicone elastomer ring device to deliver a protein vaccine formulation to the vaginal mucosal surface. Cylindrical rod-shaped inserts (2 x 7mm) were prepared by freeze-drying an aqueous hydroxypropylmethylcellulose (HPMC) gel containing recombinant CN54gp140 (500μg) with and without the TLR7/8 agonist R848 (resiquimod – 500μg). Inserts were loaded into cavities within each ring such that only the ends of the inserts were exposed. Sheep received an intramuscular injection of 100μg HIVgp140 + 200μg R848 followed by three successive ring applications of one week duration, separated by one month intervals. Other sheep received only the ring devices without priming. Serum and vaginal mucosal fluids were sampled every two weeks and analysed by CN54gp140 ELISA. Antigen-specific cellular responses were determined at necropsy.

## Results

Vaccine antigen-specific serum antibody responses were detected in both the intramuscularly primed and vaginal mucosally-primed groups. Those animals that received only vaginal vaccinations had identical IgG but superior IgA responses. Analysis revealed that all animals exhibited mucosal antigen-specific IgG and IgA with the IgA responses 30-fold greater than systemic levels. Surprisingly, very high numbers of antigen-specific B cells were detected in local genital draining lymph nodes.

## Conclusion

We have elicited local genital cellular and humoral immune responses after topical application of an adjuvanted antigen formulation within a novel vaginal ring vaccine delivery device. This regimen and delivery method elicited high levels of antigen-specific mucosal IgA and large numbers of local antigen-reactive B cells, both likely essential for effective mucosal protection.

